# First-trimester screening for fetal growth restriction and adverse maternal/perinatal outcomes

**DOI:** 10.1590/0100-3984.2025.0031

**Published:** 2025-09-01

**Authors:** Marilia de Lacerda Silva, Barbara Coppola Oliveira, Felipe Augusto Pereira dos Santos, Karine Mendonça Davi Rodrigues, Luis Ronan Marquez Ferreira de Sousa, Ana Carolina Rabachini Caetano, Edward Araujo Júnior, Luciano Marcondes Machado Nardozza, Alberto Borges Peixoto

**Affiliations:** 1 Hospital Universitário Mario Palmério – Universidade de Uberaba (UNIUBE), Uberaba, MG, Brazil; 2 Universidade Federal do Triângulo Mineiro (UFTM), Uberaba, MG, Brazil; 3 Sabin Medicina Diagnóstica, Unidade Uberaba, Uberaba, MG, Brazil; 4 Escola Paulista de Medicina – Universidade Federal de São Paulo (EPM-Unifesp), São Paulo, SP, Brazil; 5 Universidade Municipal de São Caetano do Sul (USCS), São Caetano do Sul, SP, Brazil

**Keywords:** Pregnancy trimester, first, Mass screening/methods, Fetal growth retardation/epidemiology, Pregnancy outcome/epidemiology, Aspirin/adverse effects, Primeiro trimestre da gravidez, Programas de rastreamento/métodos, Retardo do crescimento fetal/epidemiologia, Resultado do gravidez/epidemiologia, Aspirina/efeitos adversos

## Abstract

**Objective:**

To evaluate the association between first-trimester screening for fetal growth restriction (FGR) and the effect of aspirin use as prophylaxis for this condition, as well as its effect on adverse maternal and perinatal outcomes. A secondary objective was to evaluate the association between a high risk of FGR and adverse perinatal outcomes.

**Materials and Methods:**

This was a retrospective cohort study of pregnant women who did or did not undergo first-trimester screening for FGR. Screening for FGR involved the evaluation of maternal characteristics, mean arterial pressure, and the results of uterine artery Doppler. Pregnancies with an estimated risk ≥ 1:155 were categorized as high risk, whereas those with an estimated risk < 1:155 were categorized as low risk.

**Results:**

We evaluated 499 pregnant women who did not undergo first-trimester screening for FGR (unscreened group) and 615 who did (screened group). The risk of gestational hypertension was lower in the screened group, as evidenced by an adjusted odds ratio (aOR) of 0.24 (95% CI: 0.14–0.39; *p* < 0.001), as was the risk of spontaneous preterm birth at < 37 weeks of gestation (aOR: 0.22; 95% CI: 0.10–0.45; *p* < 0.001). The risk of delivery at < 32 weeks was higher in the screened group (aOR: 8.25; 95% CI: 1.05–65.71; *p* < 0.045) as was the risk of delivery at < 37 weeks (aOR: 5.91; 95% CI: 2.62–13.31; *p* < 0.001). Among all of the pregnancies at high risk of FGR (in both groups), there was an increased risk of delivery at < 32 weeks (3.1% vs. 0.2%; OR: 16.20; 95% CI: 2.20–190.90; *p* = 0.004), and at < 37 weeks (10.7% vs. 1.4%; OR: 8.41; 95% CI: 3.60–22.10; *p* < 0.0001). The use of aspirin was associated with a greater prevalence of gestational hypertension (8.0% vs. 2.1%; OR: 4.1; 95% CI: 1.77–10.10; *p* = 0.0014) and of a birth weight < 2,500 g (14.5% vs. 7.3%; OR: 2.14; 95% CI: 1.25–3.71; *p* = 0.009).

**Conclusion:**

First-trimester screening for FGR seems to be associated with a higher risk of preterm birth (at < 32 and < 37 weeks). Pregnancies that are at high risk of FGR appear to also be at a higher risk of adverse perinatal outcomes. Aspirin use seems to be associated with a greater prevalence of developing gestational hypertension and of a birth weight < 2,500 g.

## Abstract (pt)

**Objetivo:**

Avaliar a associação entre o rastreamento de primeiro trimestre para restrição de crescimento fetal (RCF) e o impacto do uso de aspirina como profilaxia para essa condição, bem como seus efeitos em resultados maternos e perinatais adversos. Avaliar a associação entre alto risco de RCF e resultados perinatais adversos.

**Materiais e Métodos:**

Estudo de coorte retrospectivo foi conduzido com gestantes que foram submetidas a triagem de primeiro trimestre ou não para RCF. A triagem para RCF foi realizada usando características maternas, pressão arterial média e Doppler das artérias uterinas. Gestantes com risco estimado ≥ 1:155 foram consideradas de alto risco, enquanto as com risco estimado < 1:155 foram consideradas de baixo risco.

**Resultados:**

Foram avaliados 499 casos que não foram submetidos a triagem de primeiro trimestre para RCF (grupo I) e 615 casos que foram submetidos a triagem de primeiro trimestre para RCF (grupo II). O grupo II apresentou menor risco de hipertensão arterial gestacional (aOR: 0,24; IC 95%: 0,14–0,39; *p* < 0,001) e parto prematuro < 37 semanas de gestação (aOR: 0,22; IC 95%: 0,10–0,45; *p* < 0,001). O grupo II apresentou maior risco de parto < 32 semanas (aOR: 8,25; IC 95%: 1,05–65,71; *p* < 0,045) e parto < 37 semanas (aOR: 5,91; IC 95%: 2,62–13,31; *p* < 0,001). Mulheres grávidas com alto risco de RCF apresentaram maior risco de parto < 32 semanas (3,1% vs. 0,2%; OR: 16,20; IC 95%: 2,20–190,90; *p* = 0,004) e parto < 37 semanas (10,7% vs. 1,4%; OR: 8,41; IC 95%: 3,60–22,10; *p* < 0,0001). O uso de aspirina foi associado a uma maior prevalência de desenvolvimento de hipertensão arterial gestacional (8,0% vs. 2,1%; OR: 4,1; IC 95%: 1,77–10,10; *p* = 0,0014) e peso ao nascer < 2.500 gramas (14,5% vs. 7,3%; OR: 2,14; IC 95%: 1,25–3,71; *p* = 0,009) em comparação com mulheres grávidas que não usaram aspirina.

**Conclusão:**

O rastreamento do primeiro trimestre para RCF foi associado a maior risco de parto < 32 semanas e parto < 37 semanas. Gestantes com alto risco para RCF apresentaram maior risco de resultados perinatais adversos. O uso de aspirina foi associado a maior prevalência de desenvolvimento de hipertensão arterial gestacional e peso ao nascer < 2.500 gramas.

## INTRODUCTION

Fetal growth restriction (FGR) is defined as the failure of the fetus to reach its intrauterine growth and developmental potential^(1)^. Affecting up to 10% of all births, FGR worsens the perinatal prognosis, making it the single largest risk factor for death in morphologically normal fetuses^(2,3)^. In addition, FGR is associated with neurological impairment in childhood and metabolic syndrome in adulthood^(4,5)^.

Currently, the most widely used classification of FGR and the one that has the greatest clinical applicability is the chronological classification. Early-onset FGR, which is usually diagnosed at < 32 weeks of gestation, is the form most often associated with preeclampsia (PE), placental damage, and umbilical artery Doppler deterioration, characteristic of fetal hemodynamic centralization. With worsening hypoxemia, abnormalities are seen on ductus venosus Doppler, as well as in the biophysical profile. Late-onset FGR usually manifests at ≥ 32 weeks of gestation and is less commonly associated with PE. In that form, in which the diffusion deficit may coexist with alterations in placental perfusion, it is common to see fetal hemodynamic centralization and altered cerebroplacental ratio with normal umbilical artery Doppler findings. Late-onset FGR is more difficult to diagnose, and fetuses with the late-onset form have a lower tolerance to hypoxia^(6)^.

To date, there is no gold standard method for diagnosing FGR^(7)^. A study of 92,218 pregnancies showed an incidence of fetal death of 9.7 cases/1,000 births when FGR was detected before delivery compared with 18.9 cases/1,000 births when it was not, demonstrating the importance of proper diagnosis and management^(3)^.

Early prediction of FGR is important because it can identify high-risk pregnant women who may benefit from preventive interventions and close monitoring during pregnancy^(8)^. Several recent studies have demonstrated the benefit of aspirin use in pregnant women at high risk for developing PE. Some of those studies evaluated the reduction in FGR rates in pregnant women at high risk of PE who received aspirin, categorizing it as a secondary effect^(9,10)^. A systematic review of the literature with a meta-analysis of 45 trials showed that the risk of FGR was reduced by almost half when aspirin was started within the first 16 weeks of gestation at a dose of 100–150 mg^(11)^.

In some countries, such as the United Kingdom and the United States, universal ultrasound examination is not recommended for assessing fetal growth in pregnant women at usual risk in their third trimester^(12)^. Recognition of pregnancies that are at high risk of FGR would be important so that the pregnant women undergo ultrasound screening for biometry and to determined the estimated fetal weight (EFW), as well as undergoing Doppler.

The aim of this study was to evaluate the association between first-trimester screening for FGR and the effect of aspirin use as prophylaxis for this condition, as well as its effect on adverse maternal and perinatal outcomes. We also evaluate the association between a high risk of FGR and adverse perinatal outcomes.

## MATERIALS AND METHODS

This was a retrospective cohort study in which data were prospectively collected between January 2020 and November 2023 from the Department of Obstetrics and Gynecology of Mario Palmério University Hospital, operated by the University of Uberaba, and from the Sabin Center for Diagnostic Medicine, both located in the city of Uberaba, Brazil. The variables were obtained from the Astraia database (Astraia Software Gmbh 2000-2015, Munich, Germany) and from electronic medical records in the Soul MV system (MV Informática Nordeste Ltda., Recife, Brazil). The study was approved by the Ethics Committee of the University of Uberaba (Reference no. 69848223.0.0000.5145). Because the data were retrospective, the requirement for informed consent was waived.

Pregnant women were divided into two groups according to whether they had been screened for FGR during the first trimester of pregnancy: the unscreened group—comprising women who had not undergone first-trimester screening for FGR involving the use of the Fetal Medicine Foundation (FMF) software; and the screened group—comprising women who had undergone first-trimester screening for FGR based on clinical criteria or involving the use of the FMF software.

The unscreened group included singleton pregnancies with gestational ages of 11–13+6 weeks, calculated from the last menstrual period or confirmed by first-trimester ultrasound at up to 13+6 weeks of gestation, in women receiving care via the primary care network of the Uberaba Municipal Department of Public Health who had not undergone clinical or combined first-trimester screening for FGR. The screened group included all singleton pregnancies with gestational ages of 11–13+6 weeks, calculated from the last menstrual period and confirmed by first-trimester ultrasound at up to 13+6 weeks of gestation, in women who had undergone first-trimester screening for FGR using the FMF software, receiving care in the Department of Fetal Medicine of Mario Palmério University Hospital or at the Sabin Center for Diagnostic Medicine. Women were excluded if a chromosomal or structural malformation was diagnosed in the fetus or in the neonate.

In Brazil, routine screening for first-trimester screening for FGR is not currently offered via the public health care system, although it is available at teaching hospitals, within the complementary health care sector, and within the private health care sector. Although some professional associations recommend biochemical screening for FGR^(9)^, that type of screening is not available at our facility. Therefore, in the present study, the screening for FGR involved a combination of taking a maternal clinical history, measuring blood pressure, and performing uterine artery (UtA) Doppler.

The individual risks of FGR with delivery at < 37 weeks of gestation were calculated by using the risk algorithm proposed by the FMF^(13)^. The variables employed to calculate the risk were maternal characteristics (e.g., age, weight, height, smoking status, chronic hypertension, preexisting diabetes mellitus, systemic lupus erythematosus, antiphospholipid syndrome, and previous pregnancy with PE), mean arterial pressure (MAP), and the mean pulsatility index (PI) on UtA Doppler.

The mean PI on UtA Doppler was measured with validated automated devices following a standardized protocol^(14)^. Color Doppler was used in order to assess the PI of the left and right UtAs on Doppler, and the mean value was recorded. All of the ultrasound examinations were performed by examiners who were certified by the FMF for Doppler evaluation of the UtAs and calculation of the risk of FGR^(15)^. Biochemical assessment, to calculate the risk of FGR by determining the concentrations of pregnancy-associated plasma protein A and placental growth factor, is not available at our facility and therefore was not performed.

The measured values of MAP and mean PI on UtA Doppler are expressed as multiples of the median on the basis of previously published equations and contained in the Astraia software^(16^–^19)^. Pregnant women in whom there was an estimated risk of PE/FGR ≥ 1:155 were considered to be at high risk, whereas those with an estimated risk < 1:155 were considered to be at low risk^(20)^. The risks were disclosed in ultrasound reports, with a recommendation to consider the use of aspirin (100 mg/day) as prophylaxis in high-risk cases. For pregnancies identified as being at high risk of FGR, the women were prescribed a daily regimen of 100 mg of aspirin, started between week 11 and week 16 of gestation and continued until week 37.

Early-onset FGR was defined as that occurring at < 32 weeks of gestation and meeting the following criteria: an EFW or abdominal circumference < the 3rd percentile for gestational age; and an EFW or abdominal circumference < the 10th percentile for gestational age, together with a mean UtA PI or an umbilical artery PI > the 95th percentile for gestational age. Late-onset FGR was defined as that occurring at ≥ 32 weeks of gestation and meeting the following criteria: an EFW or abdominal circumference < the 3rd percentile for gestational age; and an EFW or abdominal circumference < the 10th percentile for gestational age, together with an umbilical artery PI > the 95th percentile for gestational age or a cerebroplacental ratio < the 5th percentile for gestational age, with or without a drop of two quartiles in relation to the reference range^(21)^.

In the pregnant women in our study sample, the following variables were evaluated: age, ethnicity, smoking status, alcoholism, weight, height, body mass index, type of conception (spontaneous or assisted), number of previous pregnancies, number of previous deliveries, preexisting medical conditions (chronic hypertension, diabetes mellitus, systemic lupus erythematosus, or pulmonary embolism), type of delivery, intensive care unit admission, and death. In the neonates, the following variables were evaluated: gestational age at delivery, birth weight, 1-min Apgar score, 5-min Apgar score, neonatal intensive care unit admission, requirement for oxygen therapy, and death within the first 48 h of life.

The primary outcome measures were as follows: birth weight < the 3rd percentile^(22)^, birth weight < the 10th percentile^(23)^, and birth weight < 2,500 g. Other outcome measures were aspirin use during pregnancy, gestational hypertension, preterm birth at < 32 weeks, preterm birth at < 37 weeks, 5-min Apgar score < 7.0, stillbirth, and early neonatal death (within the first 48 h of life).

The data were collected and entered into an Excel 2007 spreadsheet, after which they were analyzed with the IBM SPSS Statistics software package, version 20.0 (IBM Corp., Armonk, NY, USA) and GraphPad Prism, version 7.0 (GraphPad Software; San Diego, CA, USA). The D’Agostino-Pearson normality test was used in order to determine whether the values had a Gaussian distribution. Variables with a nonparametric distribution are presented as median and range, whereas those with a parametric distribution are presented as mean and standard deviation. Categorical variables are presented as absolute value and percentage.

The Mann-Whitney test was employed to assess the effect of variables between groups. The chi-square test was used in order to assess the association between variables within the groups. Binary logistic regression was employed to calculate the odds ratio for adverse maternal/perinatal outcomes. Maternal/perinatal outcomes in the population of women who underwent first-trimester screening (general screened population) were compared with those in the population of women who did not (general obstetric population). The effect estimates are reported as prevalence ratios with 95% confidence interval (95% CI). Forward stepwise binary logistic regression was then performed, including maternal age, chronic hypertension, and diabetes mellitus as covariates to adjust the model appropriately. No adjustments were made for the comparison between the high-risk and low-risk groups, or between aspirin use and non-use, because maternal characteristics were already taken into account in the risk calculation. For all tests, the significance level was set at *p* < 0.05.

## RESULTS

We evaluated 1,114 pregnant women: 499 in the unscreened group; and 615 in the screened group. The maternal/perinatal characteristics of the study population are shown in Table 1. In comparison with what was observed in the unscreened group, maternal age was higher in the screened group (27.0 vs. 25.0 years; *p* < 0.001), as were the 1-min Apgar score (9.0 vs. 8.0; *p* < 0.001) and the 5-min Apgar score (10.0 vs. 9.0; *p* < 0.001), whereas gestational age at delivery was lower (39.1 vs. 39.4 weeks; *p* = 0.030). The screened group also had a greater proportion of women who were White (53.7% vs. 39.9%; *p* < 0.001), had previously given birth (68.3% vs. 60.7%; *p* = 0.008), had chronic hypertension (6.5% vs. 3.4%; *p* = 0.019), had preexisting diabetes mellitus (2.0% vs. 0.4%; *p* = 0.021), had had PE in previous pregnancies (4.2% vs. 0.6%; *p* < 0.001), and had undergone cesarean section (67.7% vs. 41.3%; *p* < 0.001). The prevalence of a history of pregnancy with FGR was lower in the screened group than in the unscreened group (2.8% vs. 16.2%; *p* < 0.001).

**Table 1:** Maternal/perinatal characteristics of the studied population of pregnant women (N = 1,114), submitted or not to first-trimester screening for FGR.

Characteristic	Unscreened (n = 499)	Screened (n = 615)	*P*
Maternal age (years), median (range)	25.0 (13.0–44.0)	27.0 (14.0–45.0)	< 0.001^†^
Ethnicity, n (%)			< 0.001^‡^
White	199 (39.9)	330 (53.7)	
Black	5 (1.0)	105 (17.1)	
Mixed	272 (54.5)	175 (28.5)	
Asian	23 (4.6)	5 (0.8)	
Body mass index (kg/m^2^), median (range)	25.1 (14.2–45.7)	25.4 (14.3–49.0)	0.961^†^
Number of previous deliveries, n (%)			0.008^‡^
0	196 (39.3)	195 (31.7)	
≥ 1	303 (60.7)	420 (68.3)	
Chronic hypertension, n (%)	17 (3.4)	40 (6.5)	0.019^‡^
Systemic lupus erythematosus, n (%)	1 (0.2)	1 (0.2)	0.885^‡^
Antiphospholipid syndrome, n (%)	1 (0.2)	1 (0.2)	0.884^‡^
Preexisting diabetes mellitus, n (%)	2 (0.4)	12 (2.0)	0.021^‡^
Preeclampsia in a previous pregnancy, n (%)	3 (0.6)	26 (4.2)	< 0.001^‡^
Family (maternal) history of PE, n (%)	0 (0.0)	1 (0.2)	0.367^‡^
FGR in a previous pregnancy, n (%)	81 (16.2)	17 (2.8)	< 0.001^‡^
Current smoking, n (%)	23 (4.6)	26 (4.2)	0.869^‡^
Gestational age at delivery (weeks), median (range)	39.4 (24.9–41.9)	39.1 (25.6–44.0)	0.030^†^
Type of delivery, n (%)			< 0.001^‡^
Vaginal	293 (58.7)	198 (32.2)	
Cesarean section	206 (41.3)	413 (67.2)	
Forceps	0 (0.0)	4 (0.7)	
Birth weight (g), median (range)	3,170 (470–4,885)	3,185 (840–4,175)	0.685^†^
1-min Apgar score, median (range)	8.0 (2.0–9.0)	9.0 (1.0–10.0)	< 0.001^†^
5-min Apgar score, median (range)	9.0 (2.0–10.0)	10.0 (8.0–10.0)	< 0.001^†^

† Mann-Whitney test.

‡ Chi-square test.

Table 2 shows the rates of adverse maternal/perinatal outcomes in pregnant women who underwent first-trimester screening for FGR and in those who did not. After adjustment for confounding factors (maternal age, chronic hypertension, and diabetes mellitus), the risk of gestational hypertension was found to be lower in the screened group, with an adjusted odds ratio (aOR) of 0.24 (95% CI: 0.14–0.39; *p* < 0.001), as was the risk of spontaneous preterm birth at < 37 weeks of gestation (aOR: 0.22; 95% CI: 0.10–0.45; *p* < 0.001). However, the screened group participants were found to be at a higher risk of delivery at < 32 weeks (aOR: 8.25; 95% CI: 1.05–65.71; *p* < 0.045) and of delivery at < 37 weeks (aOR: 5.91; 95% CI: 2.62–13.31; *p* < 0.001). As shown in Table 2, first-trimester screening for FGR had no effect on the risk of a birth weight < 2,500 g (*p* = 0.066), of a birth weight < the 3rd percentile for gestational age (*p* = 0.542), or of a birth weight < the 10th percentile for gestational age (*p* = 0.796).

**Table 2:** Binary logistic regression and forward stepwise binary logistic regression analyses of adverse maternal/perinatal outcomes among pregnant women submitted or not to first-trimester screening for FGR.

Adverse maternal/perinatal outcome	Unscreened (n = 499)	Screened (n = 615)	OR (95% CI)	*P* ^†^	aOR (95% CI)	*P* ^†^
Gestational hypertension, n (%)	60 (12.0)	23 (3.7)	0.28 (0.17–0.47)	< 0.001	0.24 (0.14–0.39)	< 0.001
Delivery at < 32 weeks of gestation, n (%)	1 (0.2)	10 (1.6)	8.23 (1.05–64.5)	0.045	8.25 (1.05–65.71)	0.045
Spontaneous preterm birth at < 32 weeks of gestation, n (%)	3 (0.6)	3 (0.5)	0.81 (0.16–4.03)	0.797	0.94 (0.18–4.80)	0.948
Delivery at < 37 weeks of gestation, n (%)	7 (1.4)	52 (8.5)	6.49 (2.92–14.4)	< 0.001	5.91 (2.62–13.31)	< 0.001
Spontaneous preterm birth at < 37 weeks of gestation, n (%)	35 (7.0)	10 (1.6)	0.22 (0.10–0.45)	< 0.001	0.22 (0.10–0.45)	< 0.001
Birth weight < 2,500 g, n (%)	30 (6.0)	58 (9.4)	1.63 (1.03–2.57)	0.035	1.54 (0.97–2.44)	0.066
Birth weight < the 3rd percentile, n (%)	23 (4.6)	32 (5.2)	1.14 (0.65–1.97)	0.649	1.18 (0.68–2.06)	0.542
Birth weight < the 10th percentile, n (%)	81 (16.2)	98 (15.9)	0.97 (0.70–1.35)	0.893	1.04 (0.75–1.44)	0.796
5-min Apgar score < 7.0, n (%)	3 (0.6)	0 (0.0)	0.115 (0.00–2.24)	0.054	0.00 (0.00–∞)	0.992
Fetal death, n (%)	No data	No data	No data	—	No data	—
Neonatal death within the first 48 h of life, n (%)	1 (0.2)	1 (0.2)	0.81 (0.05–13.0)	0.882	0.59 (0.03–10.06)	0.719

† Chi-square test.

aOR: adjusted odds ratio using confounding variables (adjusted for maternal age, chronic hypertension, and diabetes mellitus).

In comparison with all of the pregnancies in the unscreened group, the pregnancies that were at high risk of FGR in the screened group were also at a higher risk of delivery at < 32 weeks of gestation (3.1% vs. 0.2%; OR: 16.20; 95% CI: 2.20–190.90; *p* = 0.004), or at < 37 weeks of gestation (10.7% vs. 1.4%; OR: 8.41; 95% CI: 3.60–22.10; *p* < 0.0001), whereas they were at a lower risk of spontaneous preterm birth at < 37 weeks (1.3% vs. 7.0%; OR: 0.17; 95% CI: 0.03–0.64; *p* = 0.0047), as illustrated in Table 3.

**Table 3:** Binary logistic regression analysis of the association between the risk of FGR and adverse maternal/perinatal outcomes among pregnant women who did and did not undergo first-trimester screening for FGR.

Adverse maternal/perinatal outcome	Screened						Unscreened (n = 499)
	High risk (≥ 1:155) (n = 159)	OR (95% CI)	*P* ^†^	Low risk (< 1:155) (n = 456)	OR (95% CI)	*P* ^†^	
Gestational hypertension, n (%)	13 (8.2)	0.65 (0.34–1.21)	0.197	10 (2.2)	0.16 (0.07–0.32)	< 0.0001	60 (12.0)
Delivery at < 32 weeks of gestation, n (%)	5 (3.1)	16.20 (2.20–190.90)	0.004	5 (0.5)	5.52 (0.76–65.2)	0.109	1 (0.2)
Spontaneous preterm birth at < 32 weeks of gestation, n (%)	0 (0.0)	0.0 (0.0–3.6)	> 0.999	3 (0.7)	1.09 (0.259–4.70)	> 0.999	3 (0.6)
Delivery at < 37 weeks of gestation, n (%)	17 (10.7)	8.41 (3.60–22.10)	< 0.0001	35 (7.7)	5.84 (2.5–13.7)	< 0.0001	7 (1.4)
Spontaneous preterm birth at < 37 weeks of gestation, n (%)	2 (1.3)	0.17 (0.03–0.64)	0.0047	8 (1.7)	0.23 (0.11–0.51)	< 0.0001	35 (7.0)
Birth weight < 2,500 g, n (%)	24 (15.1)	1.66 (0.99–2.76)	0.059	34 (7.5)	1.26 (0.75–2.12)	0.437	30 (6.4)
Birth weight < the 3rd percentile, n (%)	12 (7.6)	1.68 (0.84–3.39)	0.157	20 (4.4)	0.94 (0.51–1.71)	0.877	23 (4.6)
Birth weight < the 10th percentile, n (%)	34 (21.4)	1.40 (0.89–2.19)	0.150	64 (14.0)	0.84 (0.59–1.20)	0.367	81 (16.2)
5-min Apgar score < 7.0, n (%)	0 (0.0)	0.0 (0.0–3.62)	> 0.999	0 (0.0)	0.0 (0.0–1.26)	0.250	3 (0.6)
Fetal death, n (%)	No data	No data	—	No data	No data	—	No data
Neonatal death within the first 48 h of life, n (%)	1 (0.6)	3.15 (0.16–59.98)	0.425	0 (0.0)	0.0 (0.0–9.84)	> 0.999	1 (0.2)

† Chi-square test.

All pregnant women in whom there was a high risk of FGR after first-trimester screening used aspirin (100 mg/day) until week 37 of gestation. Table 4 shows the association between the use of aspirin and adverse maternal/perinatal outcomes in pregnant women undergoing first-trimester screening for FGR. Aspirin use was found to be associated with a greater prevalence of gestational hypertension (8.0% vs. 2.1%; OR: 4.1; 95% CI: 1.77–10.10; *p* = 0.0014) and of a birth weight < 2,500 g (14.5% vs. 7.3%; OR: 2.14; 95% CI: 1.25–3.71; *p* = 0.009). Among the pregnant women who started taking aspirin in our study sample, one of every three developed gestational hypertension and one of every 5.8 delivered an infant with a birth weight < 2,500 g. Aspirin use did not reduce the risk of delivery at < 32 weeks of gestation (*p* = 0.164), spontaneous preterm birth at < 32 weeks of gestation (*p* = 0.560), delivery at < 37 weeks of gestation (*p* = 0.150), spontaneous preterm birth at < 37 weeks of gestation (*p* = 0.731), birth weight < the 3rd percentile (*p* = 0.161), birth weight < the 10th percentile (*p* = 0.088), or neonatal death within the first 48 h of life (*p* = 0.291). There were no cases of a 5-min Apgar score < 7.0 or fetal death in either of the groups analyzed.

**Table 4:** Binary logistic regression analysis of the association between aspirin use by pregnant women and adverse maternal/perinatal outcomes after first-trimester screening for FGR.

Maternal/perinatal outcome	Aspirin		OR (95% CI)	Number needed to harm	*P* ^†^
	Yes	No			
Gestational hypertension, n (%)	14 (8.0)	9 (2.1)	4.10 (1.77–10.10)	3.0	0.0014
Delivery at < 32 weeks of gestation, n (%)	5 (2.8)	5 (1.2)	2.47 (0.8–7.6)	4.7	0.164
Spontaneous preterm birth at < 32 weeks of gestation, n (%)	0 (0.0)	3 (0.7)	0.0 (0–2.81)	3.4	0.560
Delivery at < 37 weeks of gestation, n (%)	20 (11.7)	32 (7.3)	1.58 (0.88–2.79)	9.7	0.150
Spontaneous preterm birth at < 37 weeks of gestation, n (%)	2 (1.1)	8 (1.8)	0.60 (0.12–2.47)	10.8	0.731
Birth weight < 2,500 g, n (%)	26 (14.5)	32 (7.3)	2.14 (1.25–3.71)	5.8	0.009
Birth weight < the 3rd percentile, n (%)	13 (7.3)	19 (4.4)	1.71 (0.85–3.61)	8.2	0.161
Birth weight < the 10th percentile, n (%)	62 (14.2)	36 (20.1)	1.51 (0.96–2.36)	11.0	0.088
5-min Apgar score < 7.0, n (%)	No data	No data	No data	No data	No data
Fetal death, n (%)	No data	No data	No data	No data	No data
Neonatal death within the first 48 h of life, n (%)	1 (0.6)	0 (0.0)	∞ (0.27–∞)	1.4	0.291

† Chi-square test.

## DISCUSSION

Fetuses with FGR are at increased risk of perinatal death and disability. These risks are significantly lower among cases of FGR detected prenatally than among those detected after delivery^(24,25)^. Screening for FGR by combining maternal characteristics and obstetric history with a battery of biophysical and biochemical markers at 11–13 weeks of gestation could potentially identify approximately 75% of pregnancies that result in low-birth-weight infants born before 37 weeks of gestation and 45% of those that result in low-birth-weight infants born at term^(25,26)^.

In the present study, we used only clinical and biophysical markers because the biochemical analysis is not available within the public health care system of Brazil. Rocha et al.^(27)^ developed an algorithm for the prediction of PE in the first trimester of pregnancy in a population of northeastern Brazil, using only maternal characteristics and MAP, comparing it with the algorithms of the National Institute for Clinical Excellence and the American College of Obstetricians and Gynecologists. They found that those two algorithms both had low accuracy for predicting PE in a Brazilian population and that the best algorithm was that using only maternal characteristics and MAP.

Recent FGR prediction studies have been based on combining different risk factors to improve sensitivity and specificity. In a prospective cohort study conducted with the aim of creating an algorithm to predict early-onset and late-onset FGR in the first trimester of pregnancy^(28)^, the model included maternal characteristics, MAP, PI on UtA Doppler, placental growth factor, and soluble fms-like tyrosine kinase-1 levels. That study included 9,150 pregnant women, among whom there were 462 cases of FGR (59 early-onset cases and 403 late-onset cases). The authors reported a detection rate of 86.4% for early-onset FGR and of only 66.0% for late-onset FGR, with a false positive rate of 10% for both. In the present study, we used a cutoff risk of 1:155 to be considered at high risk of FGR, based on a study that used that cutoff to predict PE in Brazil. Andrade et al.^(20)^ conducted a randomized clinical trial involving 274 nulliparous pregnant women in Brazil, evaluated at 11–13+6 weeks of gestation. The 1:155 risk cutoff showed a sensitivity of 80.0%, specificity of 57.5%, positive predictive value of 19.1%, and negative predictive value of 95.0%.

In the present study, we compared pregnant women who underwent first-trimester screening for FGR with those who did not, in terms of adverse maternal/perinatal outcomes. A recent study conducted in Australia assessed pregnancy outcomes following first-trimester screening for PE^(29)^. The authors compared the pregnant women who underwent first-trimester screening with those who received the standard of care. The authors found that PE, preterm birth, small-for-gestational-age (SGA) neonates, and low Apgar scores were less common in pregnant women who underwent first-trimester screening than in the general population of pregnant women. Pregnant women in whom there is a high risk of FGR (≥ 1:100) have been shown to be more likely to develop preterm PE than are those at low risk^(29)^. In the present study, pregnant women who underwent first-trimester screening for FGR showed lower rates of gestational hypertension and preterm birth at < 37 weeks of gestation. In comparison with the study conducted in Australia^(29)^, our study utilized a higher cutoff for identifying a high risk of FGR and did not incorporate maternal biochemistry into the first-trimester screening for FGR.

The lower risk of spontaneous delivery at < 37 weeks of gestation in pregnant women in our study sample who underwent first-trimester screening for PE and FGR is mainly explained by early identification and appropriate management of risk factors. Although it is not widely recommended, we routinely perform transvaginal cervical length measurement during the first-trimester examination. Although measuring cervical length in the first trimester may not be as effective as measuring it in the second trimester, which is more commonly used in clinical practice to predict the risk of preterm birth^(30,31)^, we believe that measuring cervical length in the first trimester may have been useful when combined with other risk factors, such as a history of preterm birth, to improve the identification of women at risk and to intervene with prophylactic measures such as the use of vaginal progesterone. In the present study, we did not evaluate whether cervical length measurement during the first trimester and the use of prophylactic measures were associated with a reduced risk of spontaneous preterm birth at < 37 weeks of gestation, and studies with appropriate designs are needed in order to better evaluate those associations.

We believe that the lower risk of gestational hypertension in women in our study sample who underwent first-trimester screening for PE and FGR can be explained by several interconnected factors, including the implementation of preventive interventions such as aspirin use in prophylactic doses, which, together with counseling on habit and lifestyle changes, has been shown to significantly reduce the incidence of preterm PE and, by extension, gestational hypertension^(32,33)^.

In the present study, pregnant women in whom there was a high risk of FGR (≥ 1:155) had a higher risk of delivery at < 32 weeks or < 37 weeks of gestation, as well as a lower risk of preterm birth at < 37 weeks. Giorgione et al.^(34)^ conducted a retrospective cohort study to predict preterm PE in first-trimester screening. In that study, pregnant women with a risk ≥ 1:50 were classified as high risk and were offered aspirin (150 mg/day) as prophylaxis. Those who delivered preterm, compared with those who delivered at term, were also more likely to be classified as high risk for preterm PE. Uteroplacental insufficiency has been associated with an increased risk of PE and FGR. Consistent with a previous study^(35)^, we believe that placental dysfunction, identifiable through biochemical markers—despite those not having been investigated in our study sample—and predicted by changes on UtA Doppler, may serve as a potential precursor to complications such as spontaneous and iatrogenic preterm birth.

A recent retrospective study, aimed at predicting late-onset FGR^(36)^, evaluated 2,746 pregnant women, 129 of whom were diagnosed with late-onset FGR. The authors assessed maternal characteristics (age, weight, height, medical history, obstetric history, parity, and conception methods), first-trimester variables (MAP, hemoglobin levels, free beta-human chorionic gonadotropin, and pregnancy-associated plasma protein A), and second-trimester variables (EFW, head circumference/abdominal circumference ratio, and the PI on UtA Doppler). In that study, the significant variables for the predictive model were as follows: maternal weight and height; maternal medical history; MAP in the first trimester; and EFW and head circumference/abdominal circumference ratio in the second trimester. The authors reported a detection rate for late-onset FGR of 51.6%, with a false positive rate of 10%. The low prediction rate for late-onset FGR was a consequence of a low incidence of placental disease and of early-onset PE in their study sample.

In the present study, all pregnant women in whom there was a high risk of FGR after first-trimester screening used aspirin at 100 mg/day until week 37 of gestation. Aspirin use was associated with a greater prevalence of gestational hypertension and with a birth weight < 2,500 g. Park et al.^(37)^ conducted a prospective cohort study to determine whether the use of aspirin after first-trimester screening in pregnant women at high risk of PE reduces the prevalence of SGA neonates. The authors found that pregnant women screened for a high risk of early-onset PE were three to four times more likely to deliver a neonate classified as SGA. They also found that pregnant women at high risk for developing PE who used aspirin did not differ significantly from those who did not, in terms of the prevalence of SGA neonates. In a study conducted in Poland, Tousty et al.^(38)^ evaluated the implementation of aspirin use after first-trimester screening in a population without chronic hypertension. In that study, pregnant women in whom there was a high risk of PE and FGR (>1:100) were given aspirin at a dose of 150 mg/day. The authors found that the rates of adverse perinatal outcomes, such as gestational hypertension, late-onset PE, FGR/SGA, and gestational diabetes mellitus, were higher in the high-risk (prophylactic aspirin) group than in the low-risk (no aspirin) group.

Our study has some limitations. First, we did not have data on adherence to aspirin use by the high-risk pregnant women. In addition, we did not evaluate biochemical markers in the first-trimester screening for FGR, and that fact might decrease the accuracy of the model. Furthermore, the FMF model does not allow the inclusion of a history of pregnancy with FGR, which is the most important risk factor for the condition^(39)^, in the maternal characteristics.

## CONCLUSION

First-trimester screening for FGR seems to be associated with a higher risk of delivery at < 32 and < 37 weeks of gestation. In addition, pregnant women in whom there is a high risk of FGR appear to be at a higher risk of adverse perinatal outcomes. Furthermore, our findings indicate that aspirin use during pregnancy is associated with a higher prevalence of gestational hypertension and of a birth weight < 2,500 g.
